# 

**Published:** 1970-04

**Authors:** 


					A"?L 1970

				

